# Corneal biomechanical properties after SMILE versus FLEX, LASIK, LASEK, or PRK: a systematic review and meta-analysis

**DOI:** 10.1186/s12886-019-1165-3

**Published:** 2019-08-01

**Authors:** Hui Guo, Seyed M. Hosseini-Moghaddam, William Hodge

**Affiliations:** 1 Department of Epidemiology and Biostatistics, Schulich School of Medicine and Dentistry, Western University, London, ON Canada; 2 Toronto General Hospital, University of Toronto, Toronto, ON Canada; 3 Department of Ophthalmology, Ivey Eye Institute, St. Joseph’s Health Care London, 268 Grosvenor St., London, ON Canada

**Keywords:** Corneal biomechanical properties, Small incision lenticule extraction, Systematic review, Meta-analysis

## Abstract

**Background:**

The aim of this study was to compare the postoperative corneal biomechanical properties between small incision lenticule extraction (SMILE) and other corneal refractive surgeries.

**Methods:**

A systematic review and meta-analysis were conducted. Articles from January 2005, to April 2019, were identified searching PubMed, EMBASE, Web of Science, and International Clinical Trials Registry Platform. Studies that compared SMILE with other corneal refractive surgeries on adult myopia patients and evaluated corneal biomechanics were included. Multiple effect sizes in each study were combined. Random-effects model was conducted in the meta-analysis.

**Results:**

Twenty-two studies were included: 5 randomized controlled trials (RCTs), 9 prospective and 6 retrospective cohort studies, and 2 cross-sectional studies. Using the combined effect of corneal hysteresis (CH) and corneal resistance factor (CRF), which were obtained from ocular response analyzer (ORA), the pooled Hedges’ g of SMILE versus femtosecond laser-assisted in situ keratomileusis (FS-LASIK) was 0.41 (95% CI, 0.00 to 0.81; *p* = 0.049; I^2^ = 78%), versus LASIK was 1.31 (95% CI, 0.54 to 2.08; *p* < 0.001; I^2^ = 77%), versus femtosecond lenticule extraction (FLEX) was − 0.01 (95% CI, − 0.31 to 0.30; *p* = 0.972; I^2^ = 20%), and versus the group of photorefractive keratectomy (PRK) and laser-assisted sub-epithelial keratectomy (LASEK) was − 0.26 (95% CI, − 0.67 to 0.16; *p* = 0.230; I^2^ = 54%). The summary score of Corvis ST (CST) after SMILE was comparable to FS-LASIK/LASIK with the pooled Hedges’ g = − 0.05 (95% CI, − 0.24 to 0.14; *p* = 0.612, I^2^ = 55%).

**Conclusions:**

In terms of preserving corneal biomechanical strength after surgeries, SMILE was superior to either FS-LASIK or LASIK, while comparable to FLEX or PRK/LASEK group based on the results from ORA. More studies are needed to apply CST on evaluating corneal biomechanics after refractive surgeries.

**Electronic supplementary material:**

The online version of this article (10.1186/s12886-019-1165-3) contains supplementary material, which is available to authorized users.

## Background

Myopia is the most common type of refractive error and has a 15 to 49% prevalence worldwide [1]. Refractive surgery is a way to correct refractive error and reduce dependence on eyeglasses or contact lenses.

Photorefractive keratectomy (PRK) was the first refractive surgery approved by the U.S. Food and Drug Administration (FDA) in 1996 [2]. After epithelial removal, an excimer laser is used to remodel the cornea [3]. The most frequent complication of PRK is postoperative pain [4]. Soon after the development of PRK, laser-assisted in situ keratomileusis (LASIK), which was approved by FDA in 1998, [5] replaced PRK and has been the predominant refractive surgery worldwide since the 1990s [6–8]. In the LASIK procedure, a lamellar corneal flap is created with a mechanical microkeratome, then the flap is lifted up and excimer laser is used to make an ablation on the underlying stromal bed. After the ablation is done, the corneal flap is repositioned on the surface of the cornea [6]. After the femtosecond laser (FS) was introduced to the market in 2002, the corneal flap can be produced by FS laser instead of a microkeratome (FS-LASIK) [9]. Laser-assisted sub-epithelial keratectomy (LASEK) is another common type of refractive surgery firstly published by Massimo Camelin in 1998 [10]. Initially, an epithelial flap is detached using a diluted alcohol solution (usually 18 to 20%) on the cornea [8]. The latter surgical procedure is the same as LASIK. In 2008, the efficacy and safety after femtosecond lenticule extraction (FLEX) were reported by Sekundo et al [11]. In the FLEX procedure, a corneal flap and a lenticule from the corneal stroma under the flap are created by the femtosecond laser. The lenticule is removed with forceps [11]. In 2011, a new procedure developed from FLEX named small incision lentiule extraction (SMILE) was reported by Shah et al., and it was approved by FDA in 2016 [12, 13]. In this technique, both the lenticule and side-cut incision are made using femtosecond laser. Different from FLEX, the lenticule is removed through a small incision rather than lifting the flap.

Corneal ectasia is one of the complications of refractive surgery [14]. Although its prevalence has been reported at between only 0.04 and 0.6%, corneal ectasia is sight-threatening and may require corneal transplantation in some severe cases [15, 16]. Corneal biomechanical property changes can occur before the diagnosis of corneal ectasia, which is characterized by changes in corneal geometric features [17]. To evaluate corneal biomechanics, the most widespread devices at the time of writing are ocular response analyzer (ORA) and Corvis ST system (CST) [18, 19]. Both of them are non-contact tonometry and share some common principle: an air pulse is produced and projects to the cornea, then a set of different variables are generated related to the cornea deformation [20].

ORA uses a Scheimflug image to measure corneal deformation and produces two main biomechanical parameters. One is corneal hysteresis (CH), which is defined as the pressures (P1 and P2) difference and represents the ability to absorb the energy from the external force [21]. This ability is primarily related to corneal viscoelastic properties [22]. The other one is corneal resistance factor (CRF), which may indicate the overall corneal resistant ability [23].

Corvis ST system applies air pulse on the cornea then observes and records the movements using a high-speed Scheimpflug video camera in real time [7]. The first air puff (A1) causes the cornea to cave inward to the highest concavity (HC) and the second application (A2) is produced before it returns outwards to the natural shape. Accordingly, deformation amplitude (vertical deformation length of corneal apex), time, and length (horizontal deformation length of corneal apex) of A1, A2, and HC are calculated along with the velocity of A1 and A2. In some version of CST, deflection amplitude (deformation amplitude corrected by whole eye movement) and deflection length (deflection length of the cornea compared with the undeformed cornea) are provided at A1, A2 and HC [24, 25].

With a growing volume of refractive surgeries worldwide, the aim of this study was to compare SMILE with other corneal refractive surgeries for myopia studying the postoperative change in corneal biomechanical properties, which are often a precursor of clinically significant ectasia.

## Methods

### Inclusion and exclusion criteria

We selected the studies which performed corneal refractive surgery on adult myopia patients. The intervention was small incision lenticule extraction (SMILE). The comparator was other corneal refractive surgeries. We focused on the corneal biomechanics measured by ORA or Corvis ST as the outcome. Regarding study design, we included randomized controlled trials (RCTs), cohort, case-control or cross-sectional studies. Only studies in English were included.

### Literature search and selection strategies

**Fig. 1:**
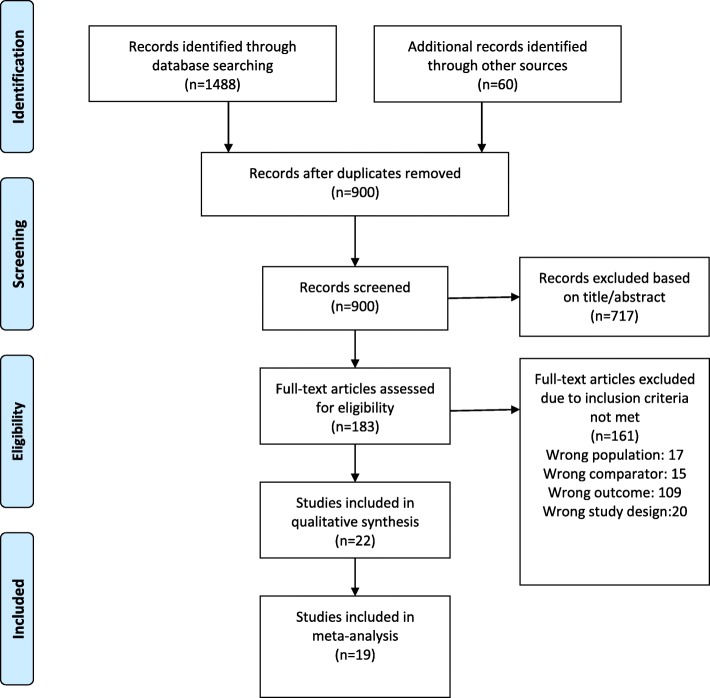
Flow Diagram of Literature Search And Study Selection

The following databases were used: PubMed, Embase, and Web of Science. The search was limited to literature published from January 01, 2005 to April 17, 2019. Search term “((((((((ora) OR ocular response analyzer) OR covis st) OR cst) OR biomechanics) OR biomechanical)) AND ((lenticule[Title/Abstract]) OR lenticules[Title/Abstract])” was applied to all the above databases. Studies that may not be published in those databases were identified by searching International Clinical Trials Registry Platform with lenticule as the search term. All the identified publications were screened independently by two authors (Hui Guo and Seyed M Hosseini-Moghaddam). Disagreements were reviewed and solved by Hui Guo, who was also responsible for data extraction. The flow chart of study selection is shown in Fig. 1 based on PRISMA guideline [26].

### Data extraction

Data extracted from the identified studies included the following information: name of the first author, year of publication, study location, surgery method, parameters of each surgery, sample size, length of follow-up, publication language, patient baseline characteristics [age, spherical equivalent (SE), central corneal thickness (CCT), and intraocular pressure (IOP) before surgery]. Regarding corneal biomechanical properties, we extracted the data including measure method, baseline value, the last follow-up value, and the change value from the baseline. If the study used ORA to measure corneal biomechanical properties, only the CH and CRF data were extracted. All the parameters achieved from CST were collected. Mean, standard deviation or standard error, and sample size were extracted for the summary measures.

### Quality assessment

We used Downs and Black checklist to assess literature quality, which includes reporting bias, external validity, information bias, selection bias, and power [27]. There are 27 questions for the five sections of assessment and a 32 score maximum. We modified the last question as to whether power and sample size were calculated and scored it 1 for “yes” answer and 0 for “no” answer [28]. Then our modified Downs and Black score ranges are given four quality levels: excellent (26–28); good (20–25); fair (15–19); and poor (≤14) [29].

### Statistical analysis

#### Imputation of variance

In the study of Li et al., [30] the mean of postoperative values of CH and CRF were reported with the absence of standard deviation (SD), standard error (SE), correlated *p*-value, or 95% confidence interval (CI). We imputed the SD using the average of SD from the other four studies in the same subgroup.

#### Within study calculation

When standard error (SE) rather than standard deviation (SD) was provided from the included studies, we computed SD = SE × $\sqrt{N}$ [31]. The effect sizes of the biomechanical outcomes achieved from ORA and CST were calculated with standardized mean difference (Hedges’ g) [32]. Then, we pooled the effect sizes and the variances of effect sizes within each study using the formula.

1 $$ \overline{Y}=\frac{1}{m}\left({\sum}_j^m{Y}_j\right) $$

and

2 $$ \mathit{\operatorname{var}}\left(\frac{1}{m}{\sum}_{i=1}^m{Y}_i\right)={\left(\frac{1}{m}\right)}^2\left({\sum}_{i=1}^m{V}_i+\sum \limits_{i\ne j}\left({r}_{ij}\sqrt{V_i}\sqrt{V_j}\right)\right) $$

with Y referring to the effect size, m to the number of outcomes, V to the variance of effect size, and r to the correlation between outcomes [33].

The correlation between CH and CRF was calculated using the weighted mean of Pearson correlation results from three studies, and we obtained an r ≈ 0.71 [24, 34, 35]. The correlation values among the outcomes from CST were obtained from the study of Bak-Nielsen et al [24]. Among each study reporting CST data, only the parameters which were reported with the correlated r were used in the meta-analysis. The composites combined from the effect sizes of CH and CRF were named CH/CRF, and those of parameters achieved from CST were named CST outcome in the following text.

Since CH and CRF have a positive correlation, we combined the effect sizes of CH and CRF directly. By contrast, the parameters from CST decreased or increased after surgeries [25, 36] and included positive and negative correlations [24]. We changed the sign of Hedges’ g by multiplying − 1 if the outcomes were negatively correlated with A1 time [37]. We also identified A1 time decreased after surgeries from previous studies [25, 36]. Examples of the combination of effect size and variance is shown in Table 1. If the study provides both postoperative and change values (postoperative values subtract preoperative values), the change values were used in the meta-analyses.

**Table 1:** Example of how to combine effect size and variance of change score of CH and CRF within studies

Study	Outcome (mmHg)	SMILE			LASIK			Effect size (Hedges’ g)	Variance of Hedges’ g	Combined effect size	Correlation between CH and CRF	Combined variance
		Mean	SD	N (eye) at last follow-up	Mean	SD	N (eye) at last follow-up					
Alper Agca [38]	CH	−1.94	1.52	30	−1.98	1.5	30	0.03	0.06	−0.07	0.71	0.06
	CRF	−2.96	1.69	30	−2.69	1.44	30	−0.17	0.07			
Di Wu [39]	CH	−1.94	0.82	37	−2.34	1.08	34	0.42	0.06	0.48	0.71	0.05
	CRF	−3.59	0.91	37	−4.29	1.6	34	0.54	0.06			
Wenjing Wu [40]	CH	−1.86	1.13	75	−2.23	1.33	75	0.3	0.03	0.4	0.71	0.02
	CRF	−3.14	1.06	75	− 3.8	1.53	75	0.5	0.03			
Bingjie Wang [41]	CH	−2.55	1.44	50	− 2.53	1.38	56	−0.01	0.04	0.4	0.71	0.03
	CRF	−2.24	1.29	50	−3.33	1.34	56	0.82	0.04			

Abbreviation: *CH* corneal hysteresis, *CRF* corneal resistance factor, *LASIK* laser-assisted in situ keratomileusis, *SD* standard deviation, *SMILE* small incision lenticule extraction

### Meta-analysis

Both the CH/CRF and CST outcomes were pooled among studies using Hedges’g. Random-effects model was selected because heterogeneity was expected due to different population and treatment regimens. Heterogeneity among studies was evaluated by *χ* ^2^ test and quantified using the I^2^ statistics [42, 43]. All reported *p*-values are 2-sided. A *p*-value equal to or less than 0.05 was considered statistically significant. Comprehensive Meta-analysis Software version 3.3.070 was used for synthesizing the outcomes among studies.

### Subgroup analysis

The meta-analysis for FS-LASIK as the comparator was divided with two subgroups based on whether follow-up time was longer than 12 months. LASEK and PRK was two separated subgroups in the comparison with SMILE. FS-LASIK and LASIK was analysed as two subgroups in the CST meta-analysis comparing SMILE and FS-LASIK/LASIK. Subgroup analyses for RCT or observational studies were conducted if applicable.

## Results

### Study identification and study characteristics

Using our search strategy, 1488 articles were identified with database searching and another 60 were identified in International Clinical Trials Registry Platform. After duplications were removed, 900 articles were reviewed for eligibility (Fig. 1). We included 22 studies in this review. Notably, we excluded one study comparing micro incision lenticule extraction and SMILE, because they are basically the same type of surgery using different incision length [44].

Five studies were RCTs, 9 were prospective cohort studies, 6 were retrospective cohort studies, and 2 were cross-sectional studies. FS-LASIK/LASIK was conducted in 15 studies, FLEX was in 3 studies, LASEK was in four studies, and PRK was included in 1 study. The length of follow-up was between 3 to 6 months in 17 studies. Four studies followed patients equal to or longer than 12 months. One study observed patients until 1 month postoperatively. Details of characters of the studies are provided in Table 2.

**Table 2:** Baseline characters of studies

First author	Publication year	Study location	Study design	Follow-up (months)	Group	N (eye) at baseline	Age (year) Mean ± SD	SE (D) Mean ± SD	CCT (*μm*) Mean ± SD	IOP (mmHg) Mean ± SD
Anders H. Vestergaard [45, 46]	2014/2019	Denmark	RCT	6	SMILE	34	35.00 ± 7.00	−7.65 ± 1.11	552.00 ± 30.00	16.10 ± 3.00
					FLEX	34	35.00 ± 7.00	−7.59 ± 0.97	553.00 ± 28.00	15.80 ± 2.80
Danyang Wang [47]	2014	China	Prospective cohort	3	SMILE (SE ≤ −6.00D)	124	24.85 ± 4.34	−4.45 ± 1.00	553.57 ± 25.50	15.75 ± 3.12
					FS-LASIK (SE ≤-6.00D)	49	25.47 ± 3.71	−4.24 ± 1.40	547.49 ± 35.00	14.79 ± 2.87
					SMILE (SE > −6.00D)	63	24.70 ± 4.68	−7.38 ± 0.95	556.00 ± 26.91	16.97 ± 2.78
					FS-LASIK (SE > −6.00D)	30	23.73 ± 3.94	−7.60 ± 1.04	539.43 ± 34.23	16.17 ± 3.23
Iben Bach Pedersen [48]	2014	Denmark	Cross-sectional	16	SMILE	29	40.90 ± 6.73	−7.10 ± 1.56	N/A	N/A
				28	FLEX	31	40.50 ± 9.47	−7.43 ± 1.11	N/A	N/A
				37	FS-LASIK	35	38.40 ± 44.55	−7.40 ± 1.18	N/A	N/A
Kazutaka Kamiya [49]	2014	Japan	RCT	3	SMILE	24	31.80 ± 6.00	−4.10 ± 1.70	543.10 ± 32.40	13.30 ± 3.20
					FLEX	24	31.80 ± 6.00	−4.10 ± 1.70	545.50 ± 31.80	13.80 ± 3.30
Di Wu [39]	2014	China	Prospective cohort	6	SMILE	40	25.75 ± 5.40	−5.71 ± 1.19	554.15 ± 24.77	N/A
					FS-LASIK	40	24.25 ± 6.02	−5.80 ± 1.14	556.70 ± 30.60	N/A
Alper Agca [38]	2014	Turkey	RCT	6	SMILE	30	26.63 ± 4.57	−3.62 ± 1.79	539.00 ± 28.00	N/A
					FS-LASIK	30	26.63 ± 4.57	−3.71 ± 1.83	542.00 ± 37.00	N/A
Yang Shen [50]	2014	China	Cross-sectional	3	SMILE	17	27.06 ± 6.77	−6.48 ± 1.22	557.65 ± 22.56	N/A
					LASEK	18	22.89 ± 6.42	−6.09 ± 1.87	533.06 ± 29.38	N/A
					FS-LASIK	17	29.53 ± 7.42	−8.71 ± 2.02	562.71 ± 20.96	N/A
Rui Dou [51]	2015	China	Retrospective cohort	3	SMILE	36	24.00 ± 8.07	−3.87 ± 0.95	538.00 ± 20.60	15.64 ± 2.04
					LASEK	35	23.00 ± 3.36	−3.51 ± 1.21	532.00 ± 32.40	15.99 ± 3.50
Shervin Mir Mohi Sefat [25]	2015	Germany	Prospective cohort	3	SMILE	43	36.60 ± 7.70	−3.81 ± 0.95	553.10 ± 29.00	15.80 ± 2.60
					FS-LASIK	26	36.20 ± 6.70	−3.65 ± 1.12	561.40 ± 30.10	15.90 ± 1.90
Wenjing Wu [40]	2015	China	Retrospective cohort	3	SMILE	75	24.25 ± 5.38	−5.49 ± 1.35	547.69 ± 27.06	15.80 ± 2.55
					FS-LASIK	75	24.28 ± 5.24	−5.56 ± 1.76	545.97 ± 27.71	15.79 ± 2.78
Hua Li [30]	2016	China	Retrospective cohort	6	SMILE	97	25.00 ± 6.00	−5.60 ± 1.43	546.75 ± 26.06	15.84 ± 2.12
					FS-LASIK	96	24.00 ± 6.00	−5.95 ± 1.78	542.86 ± 30.54	15.58 ± 2.56
Ihab Mohamed Osman [36]	2016	Egypt	Retrospective cohort	1	SMILE	25	26.28 ± 3.41	−5.43 ± 1.17	532.84 ± 16.37	14.89 ± 3.15
					LASIK	25	26.88 ± 3.99	−5.16 ± 1.42	527.96 ± 16.21	15.59 ± 3.23
Bingjie Wang [41]	2016	China	Retrospective cohort	12	SMILE	50	25.26 ± 6.64	−7.60 ± 1.12	542.96 ± 23.34	14.68 ± 2.65
					FS-LASIK	56	24.75 ± 6.24	−7.68 ± 1.19	548.00 ± 23.97	14.94 ± 2.36
Lei Xia [52]	2016	China	Prospective cohort	6	SMILE	69	25.15 ± 4.42	−5.04 ± 2.32	545.50 ± 28.20	N/A
					FS-LASIK	59	23.65 ± 3.87	−5.13 ± 1.36	538.80 ± 31.50	N/A
Minjie Chen [53]	2016	China	Prospective cohort	3	SMILE	75	26.30 ± 4.20	−4.40 ± 1.00	553.00 ± 26.50	N/A
					LASEK	76	26.70 ± 5.20	−3.70 ± 1.10	542.40 ± 34.30	N/A
Yusuf Yildirim [54]	2016	Turkey	Retrospective cohort	6	SMILE	42	29.00 ± 5.90	−3.50 ± 1.00	528.10 ± 23.60	N/A
					PRK	42	27.60 ± 5.20	−3.60 ± 0.60	517.60 ± 24.60	N/A
Jun Zhang [55]	2016	China	Prospective cohort	3	SMILE	80	N/A	−5.12 ± 1.62	550.80 ± 25.77	N/A
					FS-LASIK	80	N/A	−4.87 ± 1.80	547.06 ± 29.53	N/A
Rohit Shetty [56]	2017	India	RCT	6	SMILE	31	24.00 ± 1.00	−6.18 ± 0.41	514.18 ± 4.50	13.00 ± 0.45
					FS-LASIK	31	24.00 ± 1.00	−7.22 ± 1.32	517.00 ± 4.89	13.50 ± 0.46
Mohamed Nagy Elmohamady [57]	2018	Egypt	Prospective cohort	36	SMILE	35	24.42 ± 5.91	−8.05 ± 2.06	579.32 ± 10.65	N/A
					LASIK	30	23.84 ± 4.75	−7.49 ± 2.05	582.84 ± 12.25	N/A
					FS-LASIK	38	23.84 ± 4.75	−7.14 ± 1.97	587.96 ± 12.06	N/A
Manrong Yu [58]	2018	China	Prospective cohort	36	SMILE	32	23.40 ± 4.60	−4.10 ± 0.80	551.10 ± 23.10	17.40 ± 4.60
					LASEK	32	25.70 ± 5.70	−3.70 ± 1.00	538.30 ± 34.60	16.60 ± 2.50
Esraa El-Mayah [59]	2018	Spain	Prospective cohort	3	SMILE	30	29.53 ± 5.37	−4.17 ± 1.86	N/A	N/A
					FS-LASIK	30	27.40 ± 4.95	−3.97 ± 2.02	N/A	N/A

Abbreviations: *CCT* central corneal thickness, *CH* corneal hysteresis, *CRF* corneal resistance factor, *FLEX* femtosecond lenticule extraction, *FS* femtosecond Laser, *IOP* intraocular pressure, *LASEK* laser-assisted subepithelial keratectomy, *LASIK* laser-Assisted in situ keratomileusis, *N/A* not available, *PRK* photorefractive keratectomy, *RCT* randomized controlled trial, *SD* standard deviation, *SE* spherical equivalent, *SMILE* small incision lenticule extraction

### Surgical parameters

#### SMILE

Seventeen studies reported a cap thickness between 100 to 120 *μ* m [25, 30, 39–41, 45, 47–53, 55, 56, 58, 59]. Only one study reported a 90 *μ* m thickness cap [36]. The cap diameter was between 7.2 to 8 mm in 16 studies, [25, 30, 36, 38, 41, 45–51, 53, 58–60] and the diameter of the optical zone was between 6 to 7 mm in 19 studies [25, 30, 36, 38–41, 45, 47–49, 51–56, 58, 59]. Twelve studies were performed with an energy between 115 to 190 nJ [30, 36, 38, 40, 41, 45, 47, 49–51, 53, 54, 58].

#### LASIK

All the 14 studies [25, 30, 38–41, 47, 48, 50, 52, 55–57, 59] performed FS-LASIK except for the study of Osman et al. and Elmohamady et al., [36, 57] in which microkeratome is used for the flap creation. The flap thickness was between 90 to 110 *μm* among the 14 studies which performed LASIK [25, 30, 36, 39–41, 47, 48, 50, 52, 55–57, 59]. Eleven studies reported a flap diameter of 7.3 to 9 mm, [25, 30, 36, 38–40, 47, 48, 50, 52, 56] and the optical zone was between 5.75 to 6.75 mm in another 11 studies [25, 36, 38–41, 47, 48, 52, 55, 56]. The energy was described in 6 studies with 110 to 175 nJ [30, 38, 41, 47, 50, 52].

#### FLEX

Four studies included FLEX as a comparison treatment [45, 46, 48, 49]. In those four studies, the flap thickness was between 100 to 120 *μ* m with 7.5 to 7.9 mm in diameter and the diameter of lenticule was between 6 to 6.5 mm. Energy setting was reported in two studies with 125 to 170 nJ [45, 49].

#### LASEK

Two of the four studies which involved LASEK as the comparator reported an 8.5 mm flap diameter in 2 studies [50, 53] and optical zone was 6.25 to 6.75 mm in 1 study [58] with and the energy for ablation of 150 nJ in all 3 studies [50, 53, 58].

#### PRK

One study performed PRK as comparative surgery [54]. The optical zone was 6.5 mm. Following the PRK surgery, 0.02% mitomycin C was applied on the eyes.

### ORA and CST outcome

The data from ORA and CST measurement prepared for meta-analysis are shown in Table 3 and Table 4.

**Table 3:** Data from ocular response analyzer (ORA) measurement

First author	Procedure	N (eye) at last follow-up	Preoperative CH (mmHg)	Postoperative CH (mmHg)	CH change (mmHg)	Preoperative CRF (mmHg)	Postoperative CRF (mmHg)	CRF change (mmHg)
			Mean ± SD	Mean ± SD	Mean ± SD	Mean ± SD	Mean ± SD	Mean ± SD
Anders H. Vestergaard [45, 46]	SMILE	34	11.00 ± 1.70	7.80 ± 1.30	−3.30 ± 1.20	10.90 ± 1.90	6.40 ± 1.40	−4.60 ± 1.20
	FLEX	34	10.80 ± 1.70	8.00 ± 1.10	−2.70 ± 1.30	10.90 ± 1.80	6.40 ± 1.40	−4.50 ± 1.20
Danyang Wang [47]	SMILE(SE ≤ -6.00D)	124	10.56 ± 1.89	N/A	N/A	10.48 ± 1.89	N/A	N/A
	FS-LASIK (SE ≤ -6.00D)	49	10.45 ± 1.33	N/A	N/A	10.07 ± 1.40	N/A	N/A
	SMILE (SE > −6.00D)	63	10.49 ± 1.51	N/A	N/A	10.86 ± 1.59	N/A	N/A
	FS-LASIK (SE > −6.00D)	30	10.15 ± 1.48	N/A	N/A	10.15 ± 1.70	N/A	N/A
Iben Bach Pedersen [48]	SMILE	29	N/A	8.56 ± 1.02	N/A	N/A	7.12 ± 1.24	N/A
	FLEX	31	N/A	8.48 ± 1.00	N/A	N/A	7.00 ± 1.22	N/A
	FS-LASIK	35	N/A	8.58 ± 0.89	N/A	N/A	7.12 ± 1.06	N/A
Kazutaka Kamiya [49]	SMILE	24	10.50 ± 1.30	8.50 ± 1.00	N/A	10.00 ± 1.70	7.10 ± 1.30	N/A
	FLEX	24	10.40 ± 1.60	8.30 ± 1.10	N/A	9.80 ± 1.70	6.70 ± 1.40	N/A
Di Wu [39]	SMILE	37^b^	N/A	8.59 ± 1.00	−1.94 ± 0.82	N/A	7.78 ± 1.03	−3.59 ± 0.91
	FS-LASIK	34^b^	N/A	8.11 ± 0.66	−2.34 ± 1.08	N/A	6.94 ± 0.66	−4.29 ± 1.60
Alper Agca [38]	SMILE	30	10.89 ± 1.79	8.95 ± 1.47	−1.94 ± 1.52	10.73 ± 1.71	7.77 ± 1.37	−2.96 ± 1.69
	FS-LASIK	30	11.00 ± 1.53	9.02 ± 1.27	−1.98 ± 1.50	10.76 ± 1.45	8.07 ± 1.26	−2.69 ± 1.44
Rui Dou [51]	SMILE	36	10.00 ± 0.82	8.51 ± 0.84	−1.48 ± 0.80	10.10 ± 0.68	7.61 ± 0.83	−2.49 ± 0.71
	LASEK	35	9.99 ± 1.31	8.47 ± 1.29	−1.52 ± 1.23	10.21 ± 1.72	7.53 ± 1.42	−2.68 ± 1.03
Wenjing Wu [40]	SMILE	75	10.16 ± 1.30	8.30 ± 1.04	−1.86 ± 1.13	10.39 ± 1.52	7.25 ± 1.31	−3.14 ± 1.06
	FS-LASIK	75	10.09 ± 1.38	7.86 ± 1.03	−2.23 ± 1.33	10.57 ± 1.64	6.77 ± 1.13	−3.80 ± 1.53
Hua Li [30]	SMILE	44^b^	10.16 ± N/A	7.94 ± 1.07^a^	N/A	10.41 ± N/A	6.83 ± 1.18^a^	N/A
	FS-LASIK	38^b^	10.32 ± N/A	7.84 ± 0.88^a^	N/A	10.74 ± N/A	6.58 ± 1.01^a^	N/A
Ihab Mohamed Osman [36]	SMILE	25	12.03 ± 1.76	9.99 ± 1.76	N/A	11.42 ± 1.68	9.43 ± 1.55	N/A
	LASIK	25	11.59 ± 1.86	8.46 ± 1.76	N/A	11.00 ± 1.89	7.45 ± 2.39	N/A
Bingjie Wang [41]	SMILE	50	10.52 ± 1.71	7.97 ± 2.05	−2.55 ± 1.44	10.07 ± 1.49	7.83 ± 1.64	−2.24 ± 1.29
	FS-LASIK	56	10.85 ± 1.19	8.31 ± 1.62	−2.53 ± 1.38	10.62 ± 1.81	7.29 ± 1.76	−3.33 ± 1.34
Lei Xia [52]	SMILE	69	10.99 ± 1.65	8.58 ± 1.40	N/A	11.26 ± 1.94	7.05 ± 1.65	N/A
	FS-LASIK	59	10.76 ± 1.67	7.97 ± 1.14	N/A	10.60 ± 1.99	6.31 ± 1.41	N/A
Minjie Chen [53]	SMILE	67^b^	10.40 ± 1.70	8.30 ± 1.20	−2.20 ± 1.40	11.00 ± 1.70	7.00 ± 1.20	−4.10 ± 1.40
	LASEK	66^b^	10.00 ± 1.20	7.70 ± 1.20	−2.20 ± 1.20	10.30 ± 1.40	7.00 ± 1.50	−3.30 ± 1.00
Yusuf Yildirim [54]	SMILE	42	10.90 ± 1.70	8.40 ± 1.50	−2.50 ± 1.10	11.10 ± 1.50	7.90 ± 1.60	−3.30 ± 1.10
	PRK	42	10.40 ± 1.30	8.50 ± 1.30	−1.90 ± 1.20	10.80 ± 1.10	7.40 ± 1.50	−2.70 ± 1.10
Jun Zhang [55]	SMILE	80	10.64 ± 1.09	7.91 ± 0.92	N/A	10.54 ± 1.53	7.07 ± 1.27	N/A
	FS-LASIK	80	10.83 ± 1.60	8.00 ± 1.32	N/A	10.71 ± 1.74	6.82 ± 1.40	N/A
Mohamed Nagy Elmohamady [57]	SMILE	35	10.58 ± 0.39	8.51 ± 0.51	N/A	10.21 ± 0.09	8.38 ± 0.59	N/A
	LASIK	30	10.62 ± 0.53	7.58 ± 0.71	N/A	10.19 ± 0.12	7.17 ± 0.68	N/A
	FS-LASIK	38	10.71 ± 0.47	7.60 ± 0.61	N/A	10.22 ± 0.10	7.25 ± 0.69	N/A
Manrong Yu [58]	SMILE	32	10.50 ± 2.10	8.70 ± 1.40	N/A	11.10 ± 1.70	7.40 ± 1.10	N/A
	LASEK	32	10.10 ± 1.30	8.80 ± 1.50	N/A	10.20 ± 1.60	7.20 ± 1.70	N/A
Esraa El-Mayah [59]	SMILE	30	8.85 ± 1.80	7.37 ± 1.29	−1.44 ± 1.65	8.53 ± 2.26	6.03 ± 1.63	−2.49 ± 1.74
	FS-LASIK	30	9.83 ± 1.43	7.83 ± 1.15	−1.91 ± 0.77	9.76 ± 2.17	7.40 ± 1.35	−2.33 ± 1.27

Abbreviations: *CH* corneal hysteresis, *CRF* corneal resistance factor, *FLEX* femtosecond lenticule extraction, *FS* femtosecond Laser, *LASEK* laser-assisted subepithelial keratectomy, *LASIK* laser-Assisted in situ keratomileusis, *N/A* not available, *PRK* photorefractive keratectomy, *SMILE* small incision lenticule extraction. ^a^ the value of SD was imputed from the other four studies in the same subgroup. ^b^ The number of patients at the last follow-up visit differed from the number at baseline

**Table 4:** Postoperative outcomes of Corvis ST (CST)

First author	Iben Bach Pedersen [48]			Yang Shen [50]			Sherivin Mir Mohi Sefat [25]		Ihab Mohamed Osman [36]	
	SMILE	FLEX	FS-LASIK	SMILE	LASEK	FS-LASIK	SMILE	FS-LASIK	SMILE	LASIK
N (eye) at last follow-up	29	31	35	17	18	17	43	26	25	25
	Mean ± SD	Mean ± SD	Mean ± SD	Mean ± SD	Mean ± SD	Mean ± SD	Mean ± SD	Mean ± SD	Mean ± SD	Mean ± SD
A1 time (ms)	6.75 ± 0.16	6.76 ± 0.17	6.82 ± 0.12	7.27 ± 0.20	7.35 ± 0.23	7.17 ± 0.17	6.79 ± 0.24	6.83 ± 0.18	8.23 ± 0.37	7.89 ± 0.44
A1 deflection length (mm)	1.91 ± 0.27	1.83 ± 0.28	1.90 ± 0.24	N/A	N/A	N/A	1.97 ± 0.24	2.06 ± 0.21	N/A	N/A
A2 time (ms)	21.80 ± 0.38	21.70 ± 0.39	21.70 ± 0.35	23.08 ± 0.44	22.80 ± 0.44	22.92 ± 0.82	21.88 ± 1.11	22.05 ± 0.27	22.03 ± 1.11	20.28 ± 1.87
HC deflection amplitude (mm)	N/A	N/A	N/A	N/A	N/A	N/A	0.89 ± 0.07	0.92 ± 0.08	N/A	N/A
HC deflection length (mm)	5.93 ± 0.22	5.91 ± 0.22	5.88 ± 0.18	N/A	N/A	N/A	5.76 ± 0.22	5.82 ± 0.26	N/A	N/A
HC deformation amplitude (mm)	1.20 ± 0.05	1.18 ± 0.06	1.15 ± 0.12	1.17 ± 0.11	1.08 ± 0.11	1.19 ± 0.13	1.11 ± 0.09	1.12 ± 0.10	1.10 ± 0.08	1.26 ± 0.07
HC time (ms)	16.40 ± 0.05	16.30 ± 0.56	16.10 ± 0.47	17.38 ± 0.81	17.57 ± 0.72	17.57 ± 0.83	16.80 ± 0.36	16.77 ± 0.37	16.32 ± 1.10	14.40 ± 1.27
HC Radius (mm)	6.25 ± 0.59	6.11 ± 0.61	6.06 ± 0.53	5.74 ± 0.91	6.30 ± 1.83	6.30 ± 1.41	6.60 ± 0.70	6.60 ± 0.67	6.91 ± 1.25	7.00 ± 1.06

Abbreviations: *A* application, *FS* femtosecond laser, *HC* highest concavity, *LASEK* laser-assisted subepithelial keratectomy, *LASIK* laser-assisted in situ keratomileusis, *N/A* not available, *SD* standard deviation, *SMILE* small incision lenticule extraction. Specifically, we chose the subgroup data created in the study of Seafat et al. as this subgroup had a balance of spherical equivalent at baseline between the two intervention groups. Only one study provided the preoperative data of CST measurement. Therefore, we presented only the postoperative outcomes in this table

### Meta-analyses for ORA outcomes

**Fig. 2:**
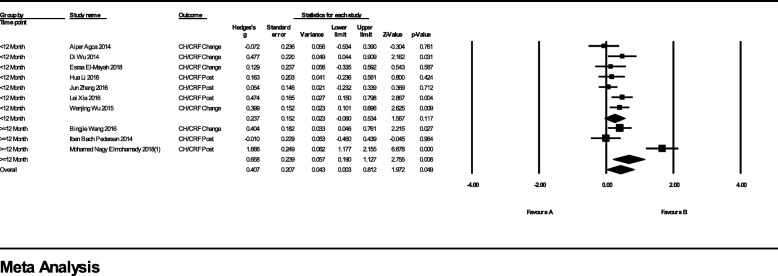
Forest Plot of Corneal Hysteresis/Corneal Resistance Factor (CH/CRF) for Studies Comparing Small Incision Lenticule Extraction (SMILE) with Femtosecond Laser-assisted in Situ Keratomileusis (FS-LASIK)

**Fig. 3:**
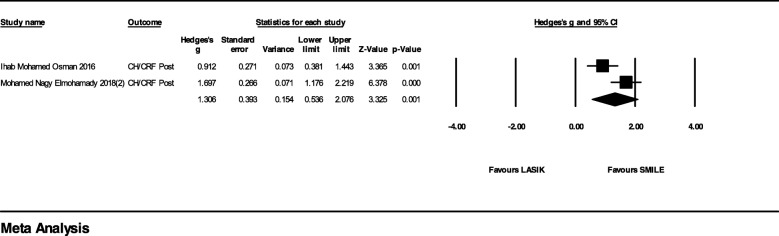
Forest Plot of Corneal Hysteresis/Corneal Resistance Factor (CH/CRF) for Studies Comparing Small Incision Lenticule Extraction (SMILE) with Laser-assisted in Situ Keratomileusis (LASIK)

In the studies with FS-LASIK as the comparator, 10 studies which provided postoperative or change value (postoperative value – preoperative value) of CH and CRF were included in the meta-analysis (Fig. 2). In the subgroup with follow-up less than 12 months, the difference of Hedges’ g between two groups was 0.24 (95% CI, − 0.06 to 0.53; *p* = 0.117; I^2^ = 25%). The difference in over 12-month follow-up subgroup was 0.66 (95% CI, 0.19 to 0.13; *p* = 0.006; I^2^ = 92%). The overall difference was 0.41 (95% CI, 0.00 to 0.81; *p* = 0.049; I^2^ = 78%). Since there is only one RCT in this meta-analysis, we conducted a subgroup analysis with observational studies only, the over-all effect size significantly favored SMILE.(Additional file 1) Compared to LASIK, SMILE also had a higher postoperative CH/CRF value with Hedges’ g = 1.31 (95% CI, 0.54 to 2.08, *p* = 0.001; I^2^ = 77%) (Fig. 3).

**Fig. 4:**
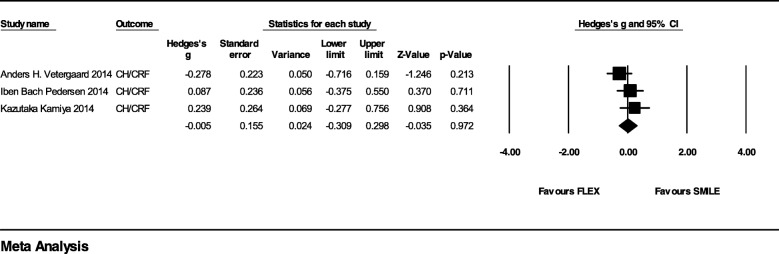
Forest Plot of Corneal Hysteresis/Corneal Resistance Factor (CH/CRF) for Studies Comparing Small Incision Lenticule Extraction (SMILE) with Femtosecond Lenticule Extraction (FLEX)

Three studies reported the CH and CRF outcomes comparing SMILE and FLEX. The effect size was almost comparable to SMILE with Hedges’ g = − 0.01 (95% CI, − 0.31 to 0.30; *p* = 0.972; I^2^ = 20%) (Fig. 4). In the subgroup analysis which included only the RCT studies of Vetergaard et al. and Kamiya et al., the difference of Hedges’ g was − 0.04 (95% CI, − 0.54 to 0.47; *p* = 0.882; I^2^ = 55%). In 2019, Vestergaard et al. used the data from the same cohort to obtain the new parameters of ORA [46]. No differences between SMILE and FLEX were found in the majority of 37 outcomes except that w11 slightly favoured FLEX.

**Fig. 5:**
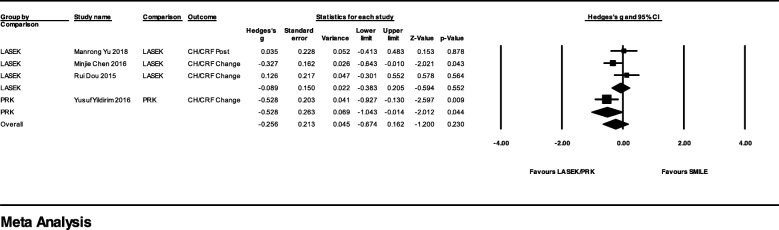
Forest Plot of Corneal Hysteresis/Corneal Resistance Factor (CH/CRF) for Studies Comparing Small Incision Lenticule Extraction (SMILE) with Laser-assisted Subepithelial Keratectomy (LASEK) /Photorefractive Keratectomy (PRK) Group

One study performed PRK, and 3 performed LASEK with ORA as the postoperative measurement. Since both PRK and LASEK remove corneal epithelium before application laser on the corneal stromal bed, and the number of the studies was too small, we pooled the effect size of these two surgeries as to compare with SMILE. Although the difference was not significant, the result showed LASEK/PRK group had a less decrease of CH/CRF after surgery than SMILE with Hedges’ g = − 0.26 (95% CI, − 0.67 to 0.16; *p* = 0.230; I^2^ = 54%). Both subgroup outcomes and overall outcomes are also provided in Fig. 5.

### Meta-analyses for CST outcomes

**Fig. 6:**
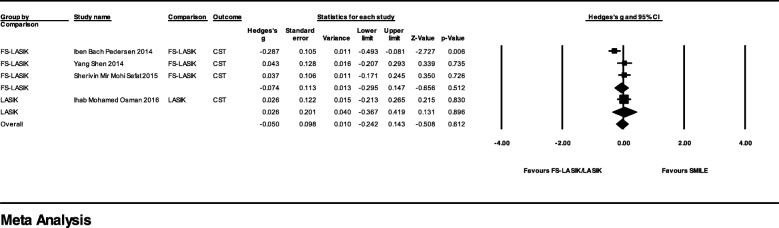
Forest Plot of Postoperative Corvis ST System (CST) Outcome for Studies Comparing Small Incision Lenticule Extraction (SMILE) with Femtosecond Laser-assisted in Situ Keratomileusis (FS-LASIK)/Laser-assisted in Situ Keratomileusis (LASIK) Group

Five studies reported corneal biomechanical outcomes with CST after FS-LASIK or LASIK. The studies and parameters that were used in the meta-analysis are shown in Table 4. The difference between SMILE and FS-LASIK was not significant with Hedges’ g = − 0.05 (95% CI, − 0.24 to 0.14; *p* = 0.612, I^2^ = 55%) (Fig. 6). Shetty et al. found both linear corneal stiffness and mean corneal stiffness obtained from CST were comparable between SMILE and FS-LASIK [56]. Since the parameters used in this study differed from the other four studies, we did not include it in the meta-analysis.

The study of Pedersen et al. [48] reported the CST outcome longer than 12 months after SMILE or FLEX. They found that eyes after both SMILE and FLEX had a significantly lower A1 deflection length compared with healthy eyes. The difference between SMILE and FLEX was not significant in HC deformation amplitude, HC radius, HC deflection length, HC time, A1 time, A1 deflection length, and A2 time.

Shen et al. [50] included LASEK as the comparator. At the 3-month postoperative follow-up, the difference between SMILE and LASEK was not significant in A1 time, HC time, A2 time, A1 length, A2 length, peak distance, A1 velocity, A2 velocity, radius, or deformation amplitude.

### Study quality assessment

In the 22 articles, the quality score ranged from 15 to 23. Eight were within good scale, and 14 were fair. Detail of quality assessment results is illustrated in Table 5.

**Table 5:** Quality checklist

No. of question	Question	Answer	Score	Anders H. Vestergaard [45, 46]	Danyang Wang [47]	Iben Bach Pedersen [48]	Kazutaka Kamiya [49]	Di Wu [39]	Alper Agca [38]	Yang Shen [50]	Rui Dou [51]	Shrvin Mir Mohi Sefat [25]	Wenjing Wu [40]	Hua Li [30]	Ihab Mohamed Osman [36]	Bingjie Wang [41]	Lei Xia [52]	Minjie Chen [53]	Yusuf Yidirim [54]	Jun Zhang [55]	Rohit Shetty [56]	Mohamed Nagy Elmohamady [57]	Manrong Yu [61]	EsraaEl-Mayah [59]
1	Is the hypothesis /aim/objective of the study clearly described?	YES	1	X	X	X	X	X	X	X	X	X	X	X	X	X	X	X	X	X	X	X	X	X
		NO	0																					
2	Are the main outcomes to be measured clearly described in the Introduction or Methods section?	YES	1	X	X	X	X	X	X	X	X	X	X	X	X	X	X	X	X	X	X	X	X	X
		NO	0																					
3	Are the characteristics of the patients included in the study clearly described?	YES	1	X	X	X	X	X	X	X	X	X	X	X	X	X	X	X	X		X	X	X	X
		NO	0																	X				
4	Are the interventions of interest clearly described?	YES	1	X	X	X	X	X	X	X	X	X	X	X	X	X	X	X	X	X	X	X	X	X
		NO	0																					
5	Are the distributions of principal confounders in each group of subjects to be compared clearly described?	YES	2		X	X	X	X	X	X	X	X	X	X	X	X	X		X		X	X	X	
		PARTIALLY	1	X														X		X				X
		NO	0																					
6	Are the main findings of the study clearly described?	YES	1	X	X	X	X	X	X	X	X	X	X	X	X	X	X	X	X		X	X	X	X
		NO	0																	X				
7	Does the study provide estimates of the random variability in the data for the main outcomes?	YES	1	X	X	X	X	X	X	X	X	X	X		X	X	X	X	X	X	X	X	X	X
		NO	0											X										
8	Have all important adverse events that may be a consequence of the intervention been reported?	YES	1			X	X			X			X		X		X	X	X	X	X	X	X	X
		NO	0	X	X			X	X		X	X		X		X								
9	Have the characteristics of patients lost to follow-up been described?	YES	1	X		X		X		X	X	X	X	X	X	X		X			X	X	X	X
		NO	0		X		X		X								X		X	X				
10	Have actual probability values been reported for the main outcomes except where the probability value is less than 0.001?	YES	1	X	X		X	X	X	X	X	X	X	X	X	X	X		X	X	X		X	X
		NO	0			X												X				X		
11	Were the subjects asked to participate in the study representative of the entire population from which they were recruited?	YES	1		X	X			X												X		X	X
		NO	0																					
		UNABLE TO DETERMINE	0	X			X	X		X	X	X	X	X	X	X	X	X	X	X		X		
12	Were those subjects who were prepared to participate representative of the entire population from which they were recruited?	YES	1																				X	
		NO	0					X	X	X														
		UNABLE TO DETERMINE	0	X	X	X	X				X	X	X	X	X	X	X	X	X	X	X	X		X
13	Were the staff, places, and facilities where the patients were treated, representative of the treatment the majority of patients receive?	YES	1		X	X		X	X	X			X	X	X	X	X	X	X	X	X			
		NO	0																				X	
		UNABLE TO DETERMINE	0	X			X				X	X										X		X
14	Was an attempt made to blind study subjects to the intervention they have received?	YES	1																					
		NO	0			X		X				X	X				X						X	X
		UNABLE TO DETERMINE	0	X	X		X		X	X	X			X	X	X		X	X	X	X	X		
15	Was an attempt made to blind those measuring the main outcomes of the intervention?	YES	1																					
		NO	0			X		X															X	X
		UNABLE TO DETERMINE	0	X	X		X		X	X	X	X	X	X	X	X	X	X	X	X	X	X		
16	If any of the results of the study were based on “data dredging”, was this made clear?	YES	1	X		X		X	X								X	X		X	X	X	X	X
		NO	0		X					X	X	X	X	X	X	X			X					
		UNABLE TO DETERMINE	0				X																	
17	In trials and cohort studies, do the analyses adjust for different lengths of follow-up of patients, or in case-control studies, is the time period between the intervention and outcome the same for cases and controls?	YES	1	X		X		X	X	X	X	X	X	X	X	X					X	X	X	X
		NO	0																					
		UNABLE TO DETERMINE	0		X		X										X	X	X	X				
18	Were the statistical tests used to asses the main outcomes appropriate?	YES	1	X	X	X	X	X	X	X	X		X		X	X	X			X	X	X	X	X
		NO	0									X		X				X	X					
		UNABLE TO DETERMINE	0																					
19	Was compliance with the inerventions reliable?	YES	1	X	X	X	X	X	X	X	X	X	X	X	X	X	X	X	X	X	X	X	X	X
		NO	0																					
		UNABLE TO DETERMINE	0																					
20	Were the main outcome measures used accurate (valid and reliable)?	YES	1	X	X	X	X	X	X	X	X	X	X	X	X	X	X	X	X	X	X	X	X	X
		NO	0																					
		UNABLE TO DETERMINE	0																					
21	Were the patients in different intervention groups (trials and cohort studies) or were the cases and controls (case-control studies) recruited from the same population?	YES	1	X	X	X	X	X	X	X	X	X	X	X	X	X	X	X	X	X	X		X	
		NO	0																					
		UNABLE TO DETERMINE	0																			X		X
22	Were study subjects in different intervention groups (trials and cohort studies) or were the cases and controls (case-control studies) recruited over the same period of time?	YES	1	X	X	X	X	X	X	X	X	X	X	X	X	X	X	X	X	X	X		X	
		NO	0																					
		UNABLE TO DETERMINE	0																			X		X
23	Were study subjects randomised to intervention groups?	YES	1	X					X												X			
		NO	0			X						X	X				X		X				X	X
		UNABLE TO DETERMINE	0		X		X	X		X	X			X	X	X		X		X		X		
24	Was the randomised intervention assignment concealed from both patients and health care staff until recruitment was complete and irrevocable?	YES	1						X															
		NO	0		X	X				X	X	X	X				X		X		X		X	X
		UNABLE TO DETERMINE	0	X			X	X						X	X	X		X		X		X		
25	Was there adequate adjustment for confounding in the analyses from which the main findings were drawn?	YES	1	X	X	X	X		X		X		X			X					X		X	
		NO	0					X		X		X		X			X	X	X	X		X		X
		UNABLE TO DETERMINE	0												X									
26	Were losses of patients to follow-up taken into account?	YES	1	X		X				X	X		X		X	X			X		X	X	X	X
		NO	0						X			X								X				
		UNABLE TO DETERMINE	0		X		X	X						X			X	X						
27	Did the study have sufficient power to detect a clinically important effect where the probability value for a difference being due to chance is less than 5%	YES	1	X		X	X		X						X									
		NO	0		X			X		X	X	X	X			X	X	X	X	X	X	X	X	X
	Total scores		28	20	17	22	17	18	22	19	18	15	20	15	20	19	17	15	16	18	23	16	21	17
	excellent (26–28); good (20–25); fair (15–19); poor (≤14)			Good	Fair	Good	Fair	Fair	Good	Fair	Fair	Fair	Good	Fair	Good	Fair	Fair	Fair	Fair	Fair	Good	Fair	Good	Fair

### Sensitivity analysis

We removed two studies from the meta-analysis for comparing SMILE and FS-LASIK. One is the study of Li et al. because the SD in this study was imputed [30]. Another one is the study of Elmohamady et al. since the effect size of the study was much higher than the rest of the studies. In this meta-analysis, the outcome was significantly favoured SMILE with Hedges’ g = 0.25 (95% CI, 0.007 to 0.08; *p* = 0.003, I^2^ = 28%) (Additional file 2).

## Discussion

To our best knowledge, this is the first systematic review and meta-analysis comparing SMILE with all the other corneal refractive surgeries in corneal biomechanical properties. We included 22 articles in this review with 19 articles in the meta-analyses.

According to the CH and CRF value measured with ORA, corneal biomechanical strength was preserved significantly better after SMILE than either FS-LASIK or LASIK. After conducting a sensitivity analysis, the result was robust after removing the possible biased data. Similarly, Yan et al. performed a meta-analysis with five studies, which are included in our meta-analysis, and reported a significant larger CH and CRF value after SMILE than FS-LASIK [62]. Furthermore, we found the difference was greater after postoperative 12 months. This might indicate wound healing is better after SMLE. By contrast, we did not find a significant difference between SMILE and FS-LASIK in the postoperative outcomes from CST. The conclusion based on CST agreed with the majority of the studies in this review [25, 48, 50, 56]. The study of Osman et al. found A1 time, A2 time, A2 length, HC time HC radius, HC peak distance, and deformation amplitude were significantly different between SMILE and LASIK group [36]. It is the only divergent study which used a microkeratome to create a corneal flap rather than femtosecond laser used in the other three studies. It might be the reason for the discrepancy of the conclusion.

In our meta-analysis, the corneal biomechanics was not statistically different between SMILE and FLEX. This conclusion agreed with the meta-analysis of Ma et al [63]. They used postoperative CH and CRF value in different subgroup analysis and pooled the results of the two subgroups. The difference between SMILE and FLEX was 0.08 mmHg (95% CI, − 0.17 to 0.33; *p* = 0.54). We found only one study comparing SMILE with FLEX in CST outcomes [48]. The study did not find a significant difference in the postoperative values between the two surgeries.

The CH/CRF value was greater after PRK/LASEK compared with SMILE although the difference did not reach a significance. In the study of Yildirim et al., the amount of stromal tissue removed by SMILE was significantly greater than PRK [54]. This may bias the result because of the greater lenticule thickness or ablation depth the more decrease of CH and CRF value after refractive surgeries [51, 54]. By contrast, Dou et al. did not find a significant difference between SMILE and LASEK in CH or CRF decrease [51]. However, the decrease of CH or CRF per unit of removed tissue was significantly smaller after SMILE than LASEK. We identified only one study comparing SMILE with LASEK in CST. No statistically significant difference between the two treatments was found in this study [50].

### Explanations for the outcome

It has been hypothesized by many authors that SMILE is superior to LASIK in preserving the biomechanical properties of corneas because of its flapless procedure [39, 47, 48, 52]. The difference between flap versus flapless procedure was also found in the study of Kamiya et al. finding that CH and CRF had a significantly greater decrease after LASIK than after PRK [64].

A vitro experimental study found that the vertical side cuts of corneal lamellae contributed more of the loss of structural integrity than horizontal delamination incisions [65]. This can explain why flap procedure is more likely to lower corneal biomechanics.

However, we found that although SMILE was better than LASIK in the outcome from ORA, SMILE was comparable to FLEX, which also included a flap-creation procedure. This may be explained by: first, the number of studies was too small to identify the difference between SMILE and FLEX; second, CH and CRF were correlated to the flap thickness.

It is possible that the thickness of the flap, which was created in the anterior lamellae was responsible for the significant decrease of CH and CRF value. In the included studies, the flap thickness in the LASIK group was between 90 to 110 *μm* and it was between 100 to 120 *μ* m in FLEX group. A laboratory study found that the anterior part of the corneal stroma (100 to 120 *μ* m) was rigid due to the tightly interwoven anterior lamellae [66]. This physiological property of cornea was approved in the vivo study from Wang et al [47]. They found that the significantly lower CH and CRF value after LASIK than SMILE was only identified in high myopia subgroup while not in low myopia subgroup. They also pointed out that the corneal flap was thinner in high myopia patients than low myopia patients treated with LASIK. It indicated that the more anterior part of stromal lamellae was affected, the more biomechanical strength was weakened.

### Limitations

There were some limitations in our study. (1) The number of studies was small, especially of the studies performing FLEX, PRK, or LASEK as comparators. (2) Only five studies in this review were RCT design. Confounders were possible to be introduced in other types of studies and bias the outcomes. (3) All the meta-analysis included no more than 10 studies, which made the test of publication bias problematic [67]. (4) The way in which we used to synthesize effect size of CH and CRF in the meta-analysis made it impossible to compare the two parameters in the efficacy of detecting the corneal biomechanical change. However, ignoring the correlation between multiple outcomes and treating the outcomes as a unit separately in the meta-analysis will overestimate the precision of the summary effects [33]. (5) High heterogeneity across studies made the mean estimate less certain in this review. It may be caused by the diverse characteristics of patients and different study design across studies. Meta-regression analysis may be the best way to address this problem. However, this method might not be applicable to such a small number of study [37]. Alternatively, we did subgroup analyses to reduce this possible bias.

### Perspective

To evaluate the impact of SMILE on corneal biomechanical properties compared with other corneal refractive surgeries, studies could be done based on several considerations. Initially, RCT would be the best study design for this scientific question, and blinding for measurement is necessary. Second, it is better to do subgroup analysis by dividing patients into low myopia and high myopia groups. Furthermore, if available, both ORA and CST measurements can be performed to evaluate the corneal biomechanical change and compare the outcomes. Longer follow-up time (more than 6 months) is necessary for better evaluation of the efficacy and safety of refractive surgery. Adverse events should be reported when publishing the study.

## Conclusions

Our results from ORA indicated that SMILE was superior to FS-LASIK/LASIK in preserving corneal biomechanical strength after surgery. SMILE versus FLEX, PRK, or LASEK regarding corneal biomechanical properties were studied in only a few trials. The biomechanical outcomes between SMILE and FLEX were comparable. Although no significant difference was found, PRK/LASEK group showed better outcomes than SMILE. CST was not sensitive in detecting the difference of postoperative corneal biomechanical properties between surgeries in our meta-analysis.

## Additional files

Additional file 1: Forest Plot of Corneal Hysteresis/Corneal Resistance Factor (CH/CRF) for Observational Studies Comparing Small Incision Lenticule Extraction (SMILE) with Femtosecond Laser-assisted in Situ Keratomileusis (FS-LASIK). (PDF 54 kb)

Additional file 2: Forest Plot of Corneal Hysteresis/Corneal Resistance Factor (CH/CRF) for Studies Comparing Small Incision Lenticule Extraction (SMILE) with Femtosecond Laserassisted in Situ Keratomileusis (FS-LASIK) from a Sensitivity Analysis. (PDF 53 kb)

## Data Availability

Data used in the analyses can be found in the published article, which were listed in the references of this manuscript.

## Abbreviations

CCT: Central corneal thickness
CH: Corneal hysteresis
CRF: Corneal resistance factor
CST: Corvis ST system
FLEX: Femtosecond lenticule extraction
FS: femtosecond laser
IOP**’**: Intraocular pressure
LASEK: Laser-assisted sub-epithelial keratectomy
LASIK: Laser-assisted in situ keratomileusis
ORA: Ocular response analyzer
PRK: Photorefractive keratectomy
RCTs: Randomized controlled trials
SE: Spherical equivalent
SMILE: Small incision lenticule extraction
