# Supplementary material for: Corneal biomechanical properties after SMILE versus FLEX, LASIK, LASEK, or PRK: a systematic review and meta-analysis
Source: BMC Ophthalmol. 2019 Aug 1;19:167. doi: 10.1186/s12886-019-1165-3 (PMC6676534; doi:10.1186/s12886-019-1165-3)
Supplement: Supplementary file 1 — Forest Plot of Corneal Hysteresis/Corneal Resistance Factor (CH/CRF) for Observational Studies Comparing Small Incision Lenticule Extraction (SMILE) with Femtosecond Laser-assisted in Situ Keratomileusis (FS-LASIK). (PDF 54 kb) [file 12886_2019_1165_MOESM1_ESM.pdf]

Appendix 1: Forest Plot of Corneal Hysteresis/Corneal Resistance Factor (CH/CRF) for Observational Studies Comparing Small Incision Lenticule Extraction (SMILE) with Femtosecond Laser-assisted in Situ Keratomileusis (FS-LASIK).

Group by Time point	Study name	Outcome	Statistics for each study						
			Hedges's g	Standard error	Variance	Lower limit	Upper limit	Z-Value	p-Value
<12 Month	Di Wu 2014	CH/CRF Change	0.477	0.220	0.049	0.044	0.909	2.162	0.031
<12 Month	Esraa El-Mayah 2018	CH/CRF Change	0.129	0.237	0.056	-0.335	0.592	0.543	0.587
<12 Month	Jun Zhang 2016	CH/CRF Post	0.054	0.146	0.021	-0.232	0.339	0.369	0.712
<12 Month	Lei Xia 2016	CH/CRF Post	0.474	0.165	0.027	0.150	0.798	2.867	0.004
<12 Month	Wenjing Wu 2015	CH/CRF Change	0.399	0.152	0.023	0.101	0.696	2.625	0.009
<12 Month			0.306	0.194	0.037	-0.073	0.686	1.581	0.114
>=12 Month	Bingjie Wang 2016	CH/CRF Change	0.404	0.182	0.033	0.046	0.761	2.215	0.027
>=12 Month	Iben Bach Pedersen 2014	CH/CRF Post	-0.010	0.229	0.053	-0.460	0.439	-0.045	0.964
>=12 Month	Mohamed Nagy Elmohamady 2018(1)	CH/CRF Post	1.666	0.249	0.062	1.177	2.155	6.678	0.000
>=12 Month			0.663	0.259	0.067	0.154	1.171	2.554	0.011
Overall			0.443	0.173	0.030	0.103	0.782	2.555	0.011

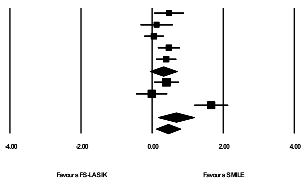

Meta Analysis
