# Supplementary material for: Corneal biomechanical properties after SMILE versus FLEX, LASIK, LASEK, or PRK: a systematic review and meta-analysis
Source: BMC Ophthalmol. 2019 Aug 1;19:167. doi: 10.1186/s12886-019-1165-3 (PMC6676534; doi:10.1186/s12886-019-1165-3)
Supplement: Supplementary file 2 — Forest Plot of Corneal Hysteresis/Corneal Resistance Factor (CH/CRF) for Studies Comparing Small Incision Lenticule Extraction (SMILE) with Femtosecond Laserassisted in Situ Keratomileusis (FS-LASIK) from a Sensitivity Analysis. (PDF 53 kb) [file 12886_2019_1165_MOESM2_ESM.pdf]

Appendix 2. Forest Plot of Corneal Hysteresis/Corneal Resistance Factor (CH/CRF) for Studies Comparing Small Incision Lenticule Extraction (SMILE) with Femtosecond Laser-assisted in Situ Keratomileusis (FS-LASIK) from a Sensitivity Analysis.

Group by Time point	Study name	Outcome	Statistics for each study						
			Hedges's g	Standard error	Variance	Lower limit	Upper limit	Z-Value	p-Value
<12 Month	Alper Agaca 2014	CH/CRF Change	-0.072	0.236	0.056	-0.534	0.390	-0.304	0.761
<12 Month	Di Wu 2014	CH/CRF Change	0.477	0.220	0.049	0.044	0.909	2.162	0.031
<12 Month	Esraa El-Mayah 2018	CH/CRF Change	0.129	0.237	0.056	-0.335	0.592	0.543	0.587
<12 Month	Jun Zhang 2016	CH/CRF Post	0.054	0.146	0.021	-0.232	0.339	0.369	0.712
<12 Month	Lei Xia 2016	CH/CRF Post	0.474	0.165	0.027	0.150	0.798	2.867	0.004
<12 Month	Wenjing Wu 2015	CH/CRF Change	0.399	0.152	0.023	0.101	0.696	2.625	0.009
<12 Month			0.258	0.097	0.009	0.067	0.448	2.647	0.008
>=12 Month	Bingjie Wang 2016	CH/CRF Change	0.404	0.182	0.033	0.046	0.761	2.215	0.027
>=12 Month	Iben Bach Pedersen 2014	CH/CRF Post	-0.010	0.229	0.053	-0.460	0.439	-0.045	0.964
>=12 Month			0.228	0.178	0.032	-0.121	0.577	1.278	0.201
Overall			0.251	0.085	0.007	0.083	0.418	2.936	0.003

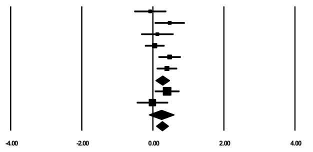

Favours FS-LASIK Favours SMILE

Meta Analysis
